# Serotonin Signaling Pathway Modulation Affects Retinal Neuron Survival in Experimental Model of Retinal Ischemia

**DOI:** 10.3390/life15111726

**Published:** 2025-11-08

**Authors:** Joanna Machowicz, Klaudia Mróz, Anna Pacwa, Anna Gąsiorek, Piotr Rodak, Joanna Lewin-Kowalik, Marialaura Amadio, Adrian Smędowski

**Affiliations:** 1 The Laboratory of Translational Research in Ophthalmology, Department of Ophthalmology, Faculty of Medical Sciences in Katowice, Medical University of Silesia, 40-752 Katowice, Poland; asiamach@interia.pl (J.M.); d201143@365.sum.edu.pl (K.M.); apacwa@sum.edu.pl (A.P.); anna.gasiorek@sum.edu.pl (A.G.); piotr.rodak@sum.edu.pl (P.R.); jlk4@wp.pl (J.L.-K.); 2 3rd Department of Cardiology, Faculty of Medical Sciences in Zabrze, Medical University of Silesia, 40-752 Katowice, Poland; 3 Department of Clinical Genetics and Rare Diseases, Faculty of Medical Sciences in Katowice, Medical University of Silesia, 40-752 Katowice, Poland; 4 GlaucoTech Co., 40-282 Katowice, Poland; 5 Department of Pediatric Neurology, Faculty of Medical Sciences in Katowice, Medical University of Silesia, 40-752 Katowice, Poland; 6 Department of Drug Sciences, University of Pavia, 27100 Pavia, Italy; marialaura.amadio@unipv.it; 7 Department of Ophthalmology, Kornel Gibinski University Clinical Center, Medical University of Silesia, 40-752 Katowice, Poland; 8 Department of Pediatric Ophthalmology, Faculty of Medical Sciences in Katowice, Medical University of Silesia, 40-752 Katowice, Poland

**Keywords:** retinal neurons, retinal ganglion cells, SSRI, ischemia, neuroprotection, electroretinography

## Abstract

Serotonin is a key neurotransmitter involved in visual processing. Selective serotonin reuptake inhibitors (SSRIs), such as Escitalopram, enhance serotonergic transmission and exert neuroprotective effects. Although these actions are well established in the central nervous system, their influence on retinal neurons remains unclear. This study investigated whether Escitalopram provides neuroprotection to retinal neurons following ischemic injury. Rats received Escitalopram or vehicle for 12 weeks. Retinal ischemia was induced by unilateral episcleral vein cauterization. A subset of animals received a retrobulbar injection of meclofenamic acid (MFA). Retinal function was assessed using electroretinography, intraocular pressure (IOP) was monitored, and retinas were collected for immunofluorescence and Western blot. Cauterization increased IOP in both groups, inducing retinal blood flow disturbances. Immunofluorescence showed a reduced number of retinal ganglion cells after cauterization, which was alleviated by SSRI treatment. Escitalopram also elevated expression of the brain-derived neurotrophic factor. Electroretinography revealed improved photopic negative response (PhNR) amplitudes in Escitalopram-treated rats, indicating improved retinal ganglion cell function. Following MFA, PhNR remained stable in SSRI-treated animals, whereas a significant impairment was observed in the vehicle-treated group. These findings demonstrate that Escitalopram provides neuroprotection by reducing both functional and structural damage in the retina and may represent a promising therapeutic strategy for retinal neurodegeneration.

## 1. Introduction

Serotonin (5-hydroxytryptamine, 5-HT) is a multifunctional biogenic monoamine produced from the essential amino acid tryptophan, which must be obtained through dietary intake. More than 90% of peripheral serotonin is synthesized in the enterochromaffin cells to modulate contractile frequency during peristalsis [1]. Importantly, although various cells can produce this neurotransmitter, the serotoninergic neurons in the central nervous system (CNS) represent the second most significant reservoir of serotonin in the human body [2]. The role of serotonin in the eye is highly complex, in part due to the coexistence of both central and peripheral neuronal elements in the mammalian visual system. In the retina, which is a part of the CNS, serotonin is synthesized and released by amacrine cells and subsequently degraded by bipolar cells and glia [3]. In addition, it has been shown that numerous serotonin receptors (5-HTR1, 5-HTR2, 5-HTR3, and 5-HTR7) are present in bipolar and ganglion cells, which may facilitate synapse formation with amacrine cells [4,5,6,7]. Within the eye, serotonin exerts a variety of effects—it modulates the processing of visual information [8,9], alters intraocular pressure (IOP) [10], and constricts ocular blood vessels [11]. Serotoninergic signaling has also been shown to exert a protective effect on the retina, as stimulation of the 5-HTR1A with an agonist protected retinal cells from light-induced photooxidative damage [12]. Serotonin plays a key role in the proper CNS functioning, and targeting its bioavailability is the main goal of antidepressants. Selective serotonin reuptake inhibitors (SSRIs) block the reuptake of serotonin into presynaptic neurons, thereby increasing extracellular serotonin levels in the synaptic cleft [13]. Moreover, SSRIs exert a range of indirect biological effects, including a reduction in nucleic acid oxidative damage [14] or the resolution of inflammation [15]. It is currently postulated that SSRIs exert pleiotropic effects on CNS. It has been demonstrated that the long-term administration of SSRIs promotes neurogenesis by enhancing neural progenitor cell (NPC) proliferation and the survival of immature neurons [16]. The mechanism of SSRIs in neurogenesis is complex. Firstly, SSRIs may directly upregulate the production of neurotrophins, particularly the brain-derived neurotrophic factor (BDNF) [17,18]. Secondly, antidepressants influence connexins (CXs)—proteins that form gap junctions, which are important during embryonic, postnatal, and adult neurogenesis [19]. CXs mediate the transmission of both pro-survival and pro-apoptotic signals between cells in CNS [19,20]. However, the functional significance of these changes has not been fully elucidated, especially regarding their effects on gap junction communication and hemichannel activity during antidepressant treatment. SSRIs are widely recognized as safe and well-tolerated drugs, offering not only therapeutic efficacy but also exhibiting neuroprotective effects. Although the beneficial effects of SSRIs on the CNS are well documented, their impact on visual neurons is poorly understood. Therefore, the presented study aimed to assess whether Escitalopram exerts neuroprotective effects on the morphology and function of retinal neurons after ischemia in a rat model. It is also worth noting that, so far, there are no effective treatments targeting post-ischemic neurodegeneration in the retina. In this study, we investigate a potential novel therapeutic strategy for ischemic diseases of the posterior segment of the eye.

## 2. Materials and Methods

### 2.1. Animals, Anesthesia, and Euthanasia

Experiments were carried out on 8-week-old male Long Evans rats, weighing approximately 200 g. All animal procedures were approved by the Local Ethical Committee for Animal Research and were in accordance with the Directive of the European Parliament (2010/63/EU). All results related to animal experiments comply with the ARRIVE (National Centre for the Replacement, Refinement & Reduction of Animal in Research) guidelines. Animals were kept under controlled environmental conditions (12 h dark/light cycle, +22 °C, 60% relative humidity), with free access to water and standard laboratory food ad libitum. Every surgical procedure was performed under general anesthesia using an intraperitoneal injection of a mixture of ketamine (50 mg/kg, VetaKetam, Vetagro, Lublin, Poland) and xylazine (5 mg/kg, Xylapan, Vetoquinol Biowet, Gorzów Wielkopolski, Poland). The animals were divided into two groups (each consisting of seven animals): an experimental group receiving Escitalopram (SSRI group) and a control group receiving phosphate-buffered saline (PBS group). The supplementation of Escitalopram was given every other day for the period of 12 weeks. After 8 weeks of continuous oral treatment, the procedure of episcleral vein cauterization (EVC) at the corneal limbus area in the right eye of every rat was performed. Furthermore, 4 weeks after this procedure, four animals from each group received a retrobulbar injection of meclofenamic acid (MFA), and three animals received a PBS injection as a control. Animals were euthanized by the intraperitoneal overdose of ketamine and xylazine followed by decapitation; then, transcardiac perfusion with PBS (500 mL) and 4% paraformaldehyde (500 mL) was performed. Tissues were collected for further retinal isolation, immunostaining, and Western blot analysis.

### 2.2. Oral Administration of SSRI

The oral administration was performed using a laboratory pipette with the tip inserted into the rat’s oral cavity. Briefly, 1 mg of Escitalopram (Betesda, Axon, Krakow, Poland) in a volume of 50 µL was administered to the animals every other day for a period of 12 weeks. Rats from the control group received 50 µL of PBS via the same procedure.

### 2.3. Retinal Ischemia Model (Episcleral Vein Cauterization)

The procedure of the episcleral vein cauterization was performed as described previously [21] under general and topical anesthesia. After 8 weeks of continuous Escitalopram treatment, the EVC was performed. The right eye of each animal was first rinsed with 10% povidone-iodine (Betadine, EGIS, Budapest, Hungary) and then with saline (Polpharma, Poland). Then, the eyeball was slightly protruded out of the orbit and four episcleral veins were cauterized using thermal cautery (Faromed, Berlin, Germany). The eye was topically treated with antibiotic ointment (Detreomycin 2%, Chema-Elektromet, Rzeszów, Poland) and protected with a sterile dressing.

### 2.4. Intraocular Pressure Measurements

The intraocular pressure (IOP) was measured in conscious animals before the procedure of episcleral vein cauterization, right after, one week and two weeks after the EVC, and then another 2 weeks later, right before the end of the experiment. IOP measurements were performed using a tonometer TonoLab (Icare, Vantaa, Finland). The cumulative IOP was calculated, defined as the sum of IOP values across consecutive measurement days.

### 2.5. Retrobulbar Injections of Meclofenamic Acid

Four weeks after the EVC procedure, the retrobulbar injection of meclofenamic acid (MFA) was performed to transiently block electrical synapses. After anesthesia and rinsing of the eyes with 10% povidone-iodine solution and saline, animals received a 3 µL retrobulbar injection of 5% MFA (Sigma Aldrich, St. Louis, MO, USA) or a PBS as a control. Injections were carried out using a 5 µL Hamilton syringe equipped with a 6 mm, 34G needle.

### 2.6. Electroretinography (ERG)

Animals were dark-adapted for 12 h before ERG testing to evaluate the retinal function under scotopic conditions. Then, systemic anesthesia with ketamine and xylazine was administered, and topical anesthesia with 0.5% proxymetacaine hydrochloride (Alcaine, Alcon, Fort Worth, TX, USA) was applied to both eyes. The pupils were dilated with 1% tropicamide (Polfa Warszawa S.A., Warsaw, Poland). During the procedure, animals were placed on a heated platform, and their eyes were moisturized with hyaluronic acid drops. ERG was recorded using a Celeris system (Diagnosys LLC, Cambridge, UK), as described previously [21,22]. The recording parameters were 0.01 cds/m^2^, 0.1 cds/m^2^, 1.0 cds/m^2^, 3.0 flash, 10 flash, and 10 Hz flicker. Retinal function was assessed using a combined dark–light-adapted protocol with measurements of a and b waves as markers of photoreceptors’ function (rods and cons), oscillatory potentials (OPs), which gave us insight into retinal interneurons, especially amacrine cells, and bipolar cells and photopic negative responses (PhNR) as a function of retinal ganglion cells (RGCs). For the analysis of OPs, we focused on OP1, OP2, and OP3, as they provide the most stable and reproducible signals and demonstrate greater sensitivity to the scotopic background light [23]. This procedure was performed at four time points: eight weeks after Escitalopram treatment—before the EVC procedure—2 weeks after the EVC procedure, again 2 weeks later—before the retrobulbar injection of MFA—and finally 30 min after injections—right before the euthanasia.

### 2.7. Immunofluorescence

Twelve weeks after the beginning of the experiment, the animals were euthanized and their retinas were collected for further analysis. Briefly, the eyeballs were removed from the orbits and placed in 3.7% formalin in PBS for 6 h at +4 °C. Then, the entire retina was isolated and placed on a microscope slide in 3.7% formalin in PBS for another 12 h at +4 °C. Subsequently, tissues were removed from the slides, placed in a 48-well plate, washed three times with PBS, and incubated with a 10% normal goat serum (NGS; Invitrogen, Carlsbad, CA, USA) in the TBST buffer (Tris-Buffered Saline with 0.1% Triton X; BioRad, Hercules, CA, USA) for 30 min to eliminate non-specific binding sites. Subsequently, retinas were cut in half and incubated overnight at +4 °C with primary antibodies—rabbit anti-RBPMS (1:500; Abcam, Cambridge, UK), mouse anti-NeuN (1:100; Sigma Aldrich, St. Louis, MO, USA), and rabbit anti-BDNF (1:100; Abcam, Cambridge, UK). After several washes with TBST buffer, retinas were incubated for three hours at room temperature (RT) with secondary antibodies—goat anti-rabbit conjugated with Alexa Fluor 488 and goat anti-mouse conjugated with Alexa 594 (1:500, Life Technologies, Waltham, MA, USA). To verify the specificity of the primary antibodies, isotype control staining was performed (Figure S1). Then, retinas were placed on microscope slides and mounted in Mowiol (Sigma Aldrich, St. Louis, MO, USA). Samples were examined under the fluorescence microscope Zeiss Axio Scope.A1 (Zeiss, Oberkochen, Germany). The mean fluorescence values were quantified with ImageJ 1.54 software (National Institutes of Health, Bethesda, MD, USA).

### 2.8. Cell Counting

RBPMS- and NeuN-positive cells were counted manually from corresponding superior and inferior quadrants of retinas using ImageJ software. From each sample, five images were captured using 200× magnification. The cells were counted within the ganglion cell layer of each retina. Then, the average number of cells per individual was calculated.

### 2.9. Western Blotting

Protein extracts from the cytoplasmic fraction of rat retinas were used for the Western blotting assay following standard procedures. After euthanasia, retinas were collected and manually homogenized. The cytoplasmic fraction was isolated using the Nuclear Extract Kit (Active Motif, Carlsbad, CA, USA), according to the manufacturer’s instructions. The protein concentration was measured with Bradford Reagent (BioRad, Hercules, CA, USA). Cytoplasmic protein samples (15 μg) were separated on 12% SDS-PAGE gels in the Mini-PROTEAN set (BioRad, Hercules, CA, USA) and transferred to polyvinylidene difluoride (PVDF) membranes (Pall Life Sciences, Portsmouth, UK) at a constant amperage of 250 mA for 90 min (Mini Trans-Blot Cell, BioRad, Hercules, CA, USA). After transfer, the PVDF membranes were blocked with 3% bovine serum albumin (BSA; Sigma Aldrich, St. Louis, MO, USA) in TBST buffer for 90 min and incubated overnight at +4 °C with the primary antibody: rabbit anti-BDNF (1:1000; Abcam, Cambridge, UK). As a housekeeping protein (loading control), an anti-tubulin antibody conjugated with rhodamine (1:3000; BioRad, Hercules, CA, USA) was used. After several washes with TBST buffer, the membranes were incubated for one hour at RT with secondary antibodies—StarBright Blue 700 goat anti-rabbit IgG (1:3000, BioRad, Hercules, CA, USA). Protein signals were detected by fluorescence (ChemiDoc MP, BioRad, Hercules, CA, USA) and densitometry analysis was performed using ImageLab 6.0.1 software (BioRad, Hercules, CA, USA).

### 2.10. Data Analysis

All results are presented as mean ± standard error of the mean (SEM) or standard deviation (SD). Statistical significance was determined using an unpaired *t*-test. Results were considered statistically significant at *p* < 0.05. The statistical analysis and data visualization were performed using GraphPad Prism 10 (GraphPad Software, LLC, San Diego, CA, USA).

## 3. Results

### 3.1. Cauterization of Episcleral Veins Effectively Increases Intraocular Pressure

**Figure 1:**
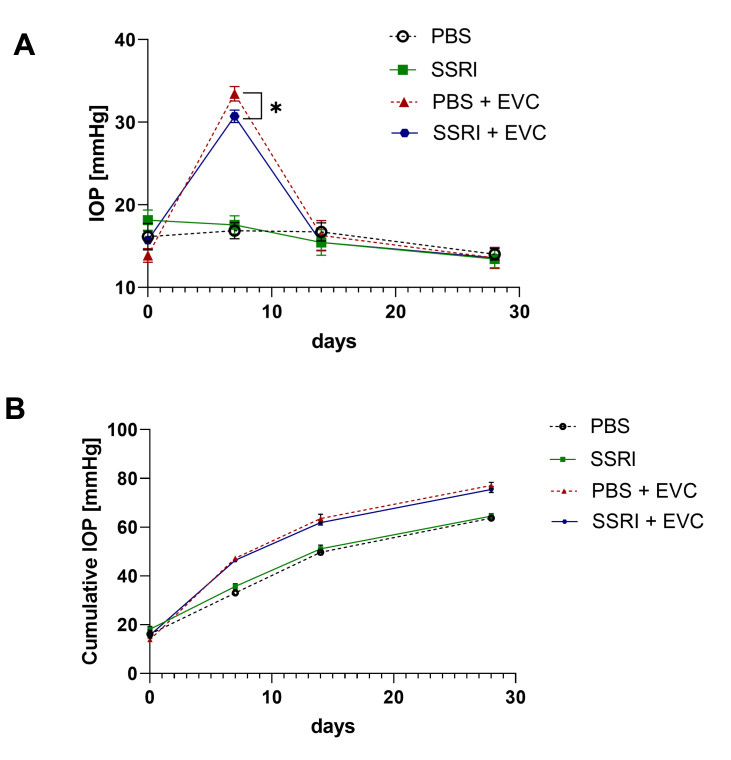
Cauterization of episcleral veins (EVCs) leads to a significant elevation in interocular pressure (IOP). (**A**) IOP values measured before cauterization (day 0); and on days 7, 14, and 28 post-EVC. (**B**) Cumulative IOP values across experimental groups. The number of animals per group *n* = 7. All data represent mean values ± standard error of the mean (SEM). *, *p* < 0.05.

The retinal ischemia model was induced by the cauterization of episcleral veins in the right eye of each rat at week eight, as described previously [21]. This procedure enables an efficient elevation of intraocular pressure to the levels that induce significant disturbances in retinal blood circulation in rodents [24,25]. The IOP values right before the procedure were comparable across all groups, not exceeding 20 mmHg. Seven days after ischemia induction, the mean IOP values in cauterized eyes from the PBS and SSRI groups increased significantly to over 30 mmHg (Figure 1A). Most notably, rats subjected to the EVC procedure and treated with Escitalopram exhibited a significantly decrease in IOP compared to the PBS-treated group on day 7; however, the cumulative IOP during the entire follow-up showed no difference between SSRI- and vehicle-treated animals (Figure 1A,B). The decline in IOP after ischemia induction was observed after 7 days and the values were stable in measurement two weeks later. Additionally, we performed IOP measurements in the control eyes, which showed no significant changes throughout the experiment.

### 3.2. Escitalopram Protects Retinal Neuronal Cells from the Deleterious Effect of Cauterization of Episcleral Veins

**Figure 2:**
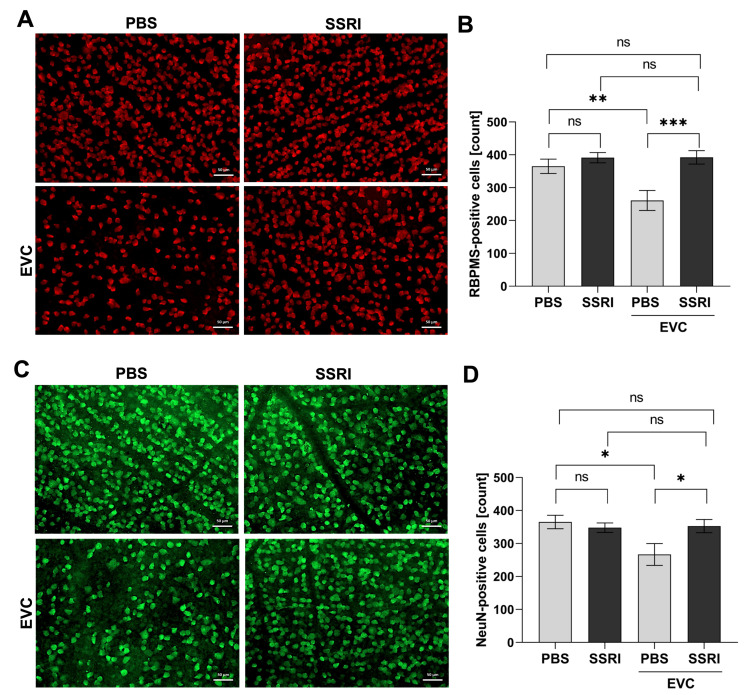
Escitalopram exerts a neuroprotective effect by preventing neuronal cell damage after the EVC procedure. Retinal tissue was stained using a specific anti-RBPMS antibody (red signal) (**A**) or an anti-NeuN antibody (green signal) (**C**) and visualized by fluorescence microscopy. Representative images were presented. Scale bar: 50 μm. Quantification of RBPMS-positive (**B**) and NeuN-positive cells (**D**). Cells were counted in the 5 fields of view in the central region of the whole-mounted retina in each sample. Then, the average number of cells per individual was calculated. The number of animals per group *n* = 3. All data represent mean value standard ± standard error of the mean (SEM). *, *p* < 0.05; **, *p* < 0.01; ***, *p* < 0.001; ns, not significant.

To assess changes in the subsequent populations of neuronal cells in the rat retina following the EVC procedure and SSRI administration, immunofluorescence staining was performed to detect the specific protein markers of these cells. RBPMS (RNA-binding protein with multiple splicing) is a selective marker of retinal ganglion cells (RGCs) [26], whereas NeuN (Neuronal Nuclei) is expressed in most CNS neurons [27]. RBPMS- (Figure 2A) and NeuN-positive (Figure 2C) cells were counted manually within the ganglion cell layer (GCL) using ImageJ software. Quantification of the fluorescence signal indicated that the number of RBPMS- and NeuN-positive cells was reduced in the retina of cauterized rats by 30% in the PBS-treated group (Figure 2B,D). However, SSRI administration reversed this effect, significantly alleviating the loss of retinal neurons after EVC to a level comparable to that observed in untreated eyes (control). Escitalopram treatment improved the survival of RBPMS-positive cells by 50% and NeuN-positive cells by approximately 30% compared with vehicle-treated rats subjected to the EVC procedure (Figure 2B,D).

### 3.3. Escitalopram Treatment Significantly Elevated BDNF Content in the Retina

**Figure 3:**
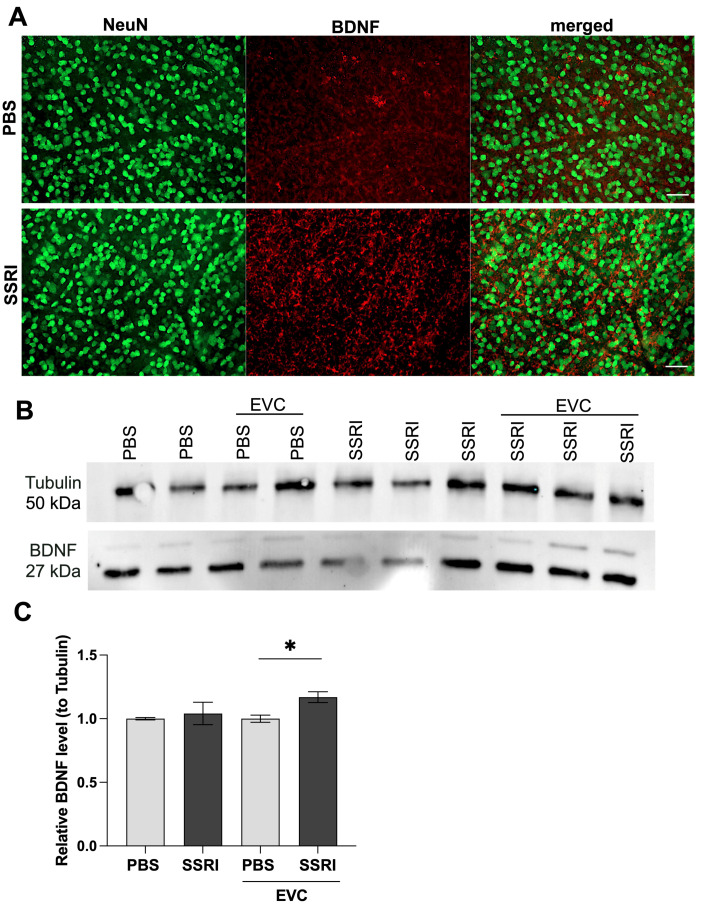
Escitalopram treatment increases BDNF content in the retina. Whole-mounted retinas were stained using a specific antibody against BDNF or NeuN, then visualized by fluorescence microscopy. (**A**) Representative images of the BDNF staining (red signal) and NeuN-positive cells (green signal) in the retina. Fluorescence was quantified in 6 fields of view. Scale bar: 50 μm. (**B**) Representative Western blot analysis of BDNF contents in protein lysates of rats’ retina. (**C**) Densitometry analysis of BDNF content presented as a fold change relative to control (PBS), normalized to tubulin levels. The number of animals per group *n* = 3. All data represent mean values ± standard error of the mean (SEM). *, *p* < 0.05.

SSRIs have been shown to induce significant changes in both behavioral functions and cellular mechanisms [28]. Therefore, to investigate whether Escitalopram treatment and the EVC procedure alter the levels of the key neuromodulator BDNF, we performed immunofluorescence staining and Western blot analysis on the cytoplasmic fraction of rat retinas. Immunostaining with the neuronal marker NeuN and specific antibody against BDNF revealed that Escitalopram treatment induces a significant increase in BDNF accumulation in the retina compared to control animals (Figure 3A). Furthermore, we found out that cauterization led to an elevation in BDNF levels in retinal homogenates, independent of SSRI administration (Figure 3B). It is noteworthy, however, that SSRI administration in cauterized rats resulted in significantly higher BDNF levels compared to rats treated with PBS (Figure 3C). These results suggest a neuroprotective role of Escitalopram during ischemia, as it increases the levels of neurotrophic factors such as BDNF, preventing further retinal damage.

### 3.4. Escitalopram Treatment Promotes Functional Recovery of the Retina

**Figure 4:**
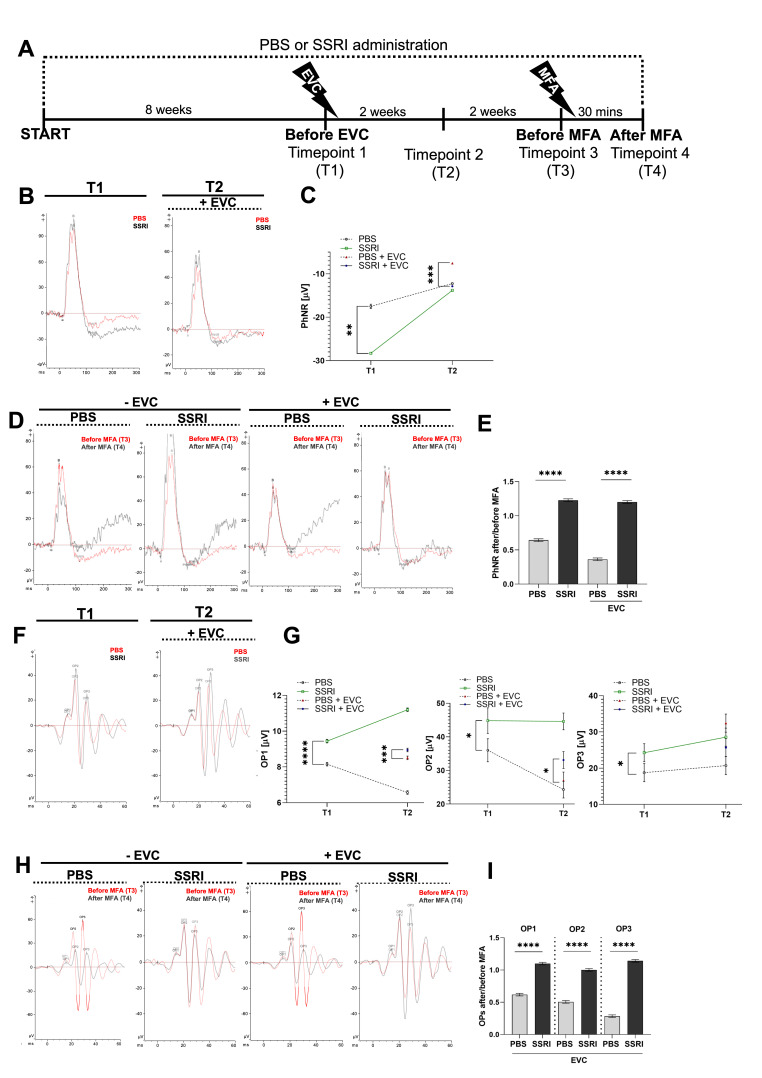
Escitalopram contributes to improvement in retinal function recovery. (**A**) Graphical representation of performed procedure. Electroretinography (ERG) was performed at four time points: T1—after 8 weeks of PBS/Escitalopram treatment; T2—2 weeks post-EVC; T3—2 weeks after T2 (before MFA injection); T4—30 min after MFA injection. Rats received PBS or Escitalopram orally throughout the experiment. (**B**) Representative ERG recordings with analysis of the photopic negative response (PhNR) wave at T1 and T2 for PBS- (red line) and SSRI-treated groups (black line). (**C**) Kinetics of PhNR changes over time. (**D**) Representative ERG recordings with analysis of PhNR wave before (T3, red line) and after (T4, black line) retrobulbar injection of MFA. (**E**) PhNR ratio after/before injection of MFA. (**F**) Representative oscillatory potentials (OPs) obtained at T1 and T2 for PBS- (red line) and SSRI-treated group (black line). (**G**) Kinetics of OP1, OP2, OP3 changes over time. (**H**) Representative OPs signal before (red line) and after (black line) retrobulbar injection of MFA. (**I**) OPs ratio after/before injection of MFA in animals subjected to EVC procedure (+EVC). The number of animals per group *n* = 3 (−MFA) or 4 (+MFA). All data represent mean values ± standard deviation (SD). *, *p* < 0.05; **, *p* < 0.01; ***, *p* < 0.001; ****, *p* < 0.0001.

To evaluate the effects of Escitalopram treatment followed by the EVC procedure on retinal function, electroretinography was performed at four experimental time points as follows: T1—after eight weeks of oral PBS or Escitalopram administration; T2—two weeks after the EVC procedure; T3—two weeks after T2 (before retrobulbar injection of MFA); and T4—30 min after a retrobulbar injection of MFA. Rats received oral administration of either PBS or Escitalopram throughout the entire experimental period (Figure 4A).

In ERG tests, the PhNR responses, which correlate with RGC function, showed significant differences between groups (Figure 4B,C). At T1, the experimental group that received Escitalopram for eight weeks had a 65% more negative PhNR amplitude compared with the PBS-treated control group, indicating an early treatment-associated alteration in retinal ganglion cells’ function. This finding suggests that SSRI treatment alone may improve the functional response of RGCs (Figure 4C). After the EVC procedure (T2), RGCs function aggravated in both groups (Figure 4B). Nevertheless, the PhNR amplitude was 62% more negative in the SSRI-treated group compared with the PBS-treated group, suggesting that Escitalopram exerts a protective effect on RGCs also under stress conditions (Figure 4C). Retrobulbar injection of MFA, an inhibitor of gap junction conductance and electrical synaptic transmission [29,30], resulted in impaired RGC function in PBS-treated animals, whereas it did not affect the PhNR amplitude in the Escitalopram-treated group (Figure 4D). Furthermore, MFA injection following the EVC procedure intensified RGC dysfunction in the PBS group (Figure 4D,E).

After 8 weeks of Escitalopram treatment (T1), differences in oscillatory potential amplitudes were observed (Figure 4F,G), particularly in OP2 and OP3, where the amplitudes were significantly higher than in the PBS-treated group. During the subsequent weeks of treatment, OP1 and OP2 amplitudes tended to rise in the Escitalopram-treated group, while in the PBS-treated group, OP1 and OP2 amplitudes tended to decline (Figure 4G). OP3 amplitude remained at a relatively stable level in both groups (T1 vs. T2). The EVC procedure inhibited the upward trend in OPs amplitudes in Escitalopram-treated animals, with the values either decreasing or remaining similar to those recorded prior to the procedure (T2). Importantly, despite the EVC procedure, OP1 and OP2 amplitudes were still higher in the SSRI-treated group compared to the PBS-treated group (Figure 4F,G). Administration of MFA led to a decrease in the amplitudes and prolonged culmination times of OPs in the PBS-treated group, both in eyes subjected to the EVC procedure and in eyes without EVC (Figure 4H). In the Escitalopram-treated group, amplitudes showed only a slight decrease and culmination times remained comparable to the initial values in eyes without EVC. Interestingly, in eyes that underwent the EVC procedure, OP amplitudes increased following Escitalopram treatment, while latencies remained the same (Figure 4H,I).

## 4. Discussion

SSRIs are considered a first-line pharmacological treatment for the spectrum of depression disorders worldwide, with their usage increasing significantly over the years. It is estimated that over 3% of the global population uses antidepressants regularly [31]. Apart from the well-established therapeutic effects in mental disorders, SSRIs also exert other biological effects, and these mechanisms are being explored in the treatment of other conditions such as neuropathic pain (neuralgia), diabetic neuropathy, irritable bowel syndrome, rheumatoid arthritis [32], lung inflammation [33], and multiple sclerosis [34]. So far, many authors presented the neuroprotective effects of Escitalopram compared to Citalopram in the suppression of neuroinflammation and reducing neuronal death [35]. Shetty et al. conducted a study regarding the protective effect of oral administration of Escitalopram in Wistar rats against the 3-NP agent, playing a crucial role in the pathophysiology of Huntington’s disease. They concluded that this particular SSRI might play a significant neuroprotective role in the management of this condition [36]. Several clinical studies have indicated that treatment with SSRIs may improve clinical recovery from acute ischemic stroke independently from depression [37]. They also improve infarct volume and neurobehavioral outcomes in animal models of ischemic stroke [38].

However, to date, there has not been a comprehensive description of how SSRIs may affect retinal function after ischemic injury; moreover, there are no effective neuroprotective treatments targeting ischemic neurodegeneration in the retina [39,40]. Ocular vascular disorders become more prevalent with age, and it still remains a common cause of blindness. Ischemia plays a key role in the pathogenesis of numerous retinal diseases, including glaucoma [41], diabetic retinopathy [42], age-related macular degeneration [43], and ocular ischemic syndrome [44]. Therefore, finding new and effective treatment methods is of high importance.

In our work, we used a well-established rat model of retinal ischemia induced by episcleral vein cauterization, which leads to the increase in the intraocular pressure that disturbs retinal blood circulation and then leads to the death of retinal neurons [21,45,46]. Importantly, the IOP levels induced in our model (>30 mmHg) have been shown to cause substantial disturbances in retinal blood flow and a decrease in the capillaries’ perfusion, as demonstrated by numerous studies employing rat models of ischemia [24,25,47]. At this stage of study, we demonstrated that SSRI exerts an IOP-independent protective effect, as rats treated with Escitalopram and PBS exhibited similar IOP following the EVC procedure. Overall, the views on the use of SSRIs and their impact on IOP remain controversial. While some data suggest that antidepressant use leads to reduction in IOP [48], the opposite effect has also been proposed [10]. RGCs are very sensitive and fragile cells to ischemia and subsequent hypoxia [49,50]. The retina is one of the most metabolically active tissues in the body and consumes oxygen even more rapidly than the brain [51]. When ischemia extends beyond one hour, it causes severe neuronal damage and often leads to vision loss [52]. Numerous mechanisms are implicated in triggering RGC apoptosis and necrosis after ischemia with the main ones proposed being increased production of reactive oxygen species [53], elevated levels of pro-inflammatory cytokines [54], and the activation of iNOS in infiltrating leukocytes or in the neurons themselves [55]. Currently, the use of SSRIs as modulators of inflammation appears quite promising, potentially contributing to their neuroprotective effects on the central nervous system. Escitalopram can reduce the levels of pro-inflammatory cytokines—IL-1β, IL-1ra, IL-2, IL-4, IL-6, IL-8, IFN-y, TNF-α in the serum of patients suffering from depression [56], while simultaneously increasing the level of anti-inflammatory IL-10 [57]. The exact mechanism behind this anti-inflammatory effect is still not fully understood; however, it is suggested that it may involve the suppression of the NF-κB signaling pathway [58], as well as direct prevention of the formation of the NRLP3 inflammasome [59]. Moreover, SSRIs increase the levels of glutathione (GSH) in the central nervous system which is one of the most important cellular free radical scavengers in the brain, thus presenting anti-oxidative properties [60]. SSRIs have been reported to hamper nitric oxide production in vivo in rats’ brains [61]. Additionally, treatment with SSRIs alters the metabolites within the kynurenine pathway. Escitalopram ameliorates neurotoxicity by increasing the levels of kynurenic acid (KYNA) with an anti-inflammatory property and decreasing the pro-inflammatory quinolinic acid (QUINA) in vitro and in vivo studies [62,63].

At the molecular level, SSRI treatment and subsequent 5-HT signaling affect the transcription of many target genes. Escitalopram activates the expression of serotonergic genes in humans and rodents, such as *Creb1* and *SLC5A4*; enzymes involved in neurotransmitter metabolism (*COMT* and *Th*), as well as neuroplasticity-related genes such as *BDNF* and *Serpini1* [64]. We demonstrated that BDNF content increased significantly in the retina following Escitalopram treatment, thus possibly protecting the neuronal tissue. BDNF is a crucial neurotrophin that plays a key role in neuronal survival, growth, and plasticity, protecting neurons under unfavorable conditions. Our results are in line with the previous research, demonstrating that pre- and post-treatment with Escitalopram increased the BDNF level in the gerbil hippocampus after ischemia [65,66]. The molecular action of BDNF in neurons is complex; it activates MAP/ERK and PI3K/Akt signaling pathways, which increases the expression of pro-survival genes [65]. Moreover, in the rat model of depression, Escitalopram elevated the BDNF levels, thereby reducing the oxidative stress in the hippocampus [67]. In the future, it may also be promising to investigate the transcriptome and proteome of retinal tissue to identify additional protein candidates activated by Escitalopram and reveal potential targets for therapeutic intervention. Among such protein candidates, connexins—particularly Connexin-36 (CX36)—are of special interest. CX36 is a gap junction protein critical in retinal neurons, and its abnormal function is implicated in retina degeneration and retinopathy [68]. It has been shown that administration of the antidepressant, fluoxetine, significantly upregulated the expression of CX36 in the hippocampus, prefrontal cortex, and amygdala in rats [69]. It is also important to note that the expression of CX36 has been shown to increase after ischemia in the murine hippocampus and this upregulation appears to exacerbate neuronal death, as gap junctions can also transfer pro-apoptotic signals [70]. Nevertheless, our preliminary research demonstrates that Escitalopram may promote the accumulation of CX36 in RGCs of rats subjected to EVC (Figure S2A,B). Interestingly, our early studies conducted in a mouse model support our findings (Figure S2C). This suggests that, under these conditions, CX36 may exert an alternative pro-survival and neuroprotective function by improving the electrical conductivity of neuronal tissue. In our study, electroretinography provides a new understanding of the effect of treatment with Escitalopram on retinal cells and function. The PhNR wave amplitude was significantly more negative in animals receiving Escitalopram, indicating improved RGC function. Potentially, Escitalopram treatment acted as a form of preconditioning, helping to protect RGCs against ischemia-induced stress during the EVC procedure. Escitalopram treatment increased the amplitudes of oscillatory potentials OP2 and OP3 in ERG recordings, suggesting enhanced synaptic activity in the inner retinal layers, primarily reflecting the activity of amacrine cells and their interactions with bipolar cells. Retrobulbar injection of MFA, which partially blocks electrical transmission trough gap junctions, prolonged OP latencies in the PBS-treated group. This observation is in line with our previous finding that MFA injection transiently blocked gap junctions and prolonged P1 wave latency [29]. However, prior treatment with Escitalopram prevented this effect, maintaining higher amplitudes and stable culmination times. These findings indicate that Escitalopram enhances inner retinal neurotransmission, preserves the function of circuits connected via gap junctions, and maintains their synchrony under ischemic conditions, suggesting a neuroprotective effect on retinal neurons and retinal neuronal syncythium. Not many studies so far have studied the effect of antidepressants on retinal function. Moulard et al. assessed the retinal function using electroretinography in patients with depression treated with different antidepressants, which showed some changes, e.g., in a reduced b-wave amplitude of photopic fERG [71]. Another review presents a novel approach to the assessment of patients with depressive disorders, where performing electroretinography measurements could serve as indicators in defining patients’ profiles, leading to more personalized therapeutic options [72]. Regarding MFA, the high dose of this substance leads to the functional and morphological damage of the retina, causing retinal toxicity and impairment of visual transduction Sun et al. revealed that MFA decreased the amplitudes of the b-wave and a-wave during ERG measurements [73].

In our study, we evaluated the impact of Escitalopram on retinal neurons during ischemic conditions. The results show that Escitalopram exerts neuroprotective activities by, among others, decreasing functional and morphological damage to the retina. These data are consistent with available scientific evidence; however, the data for using Escitalopram in retinal diseases is definitely lacking.

Observations from this study may lead to further testing and extension of Escitalopram clinical indications as a drug not only for the treatment of depression but also for retinal ischemic conditions, where it could potentially slow the progression of the disorder or even enhance the function of the neurons within the damage already present.

## Data Availability

The data presented in this study are available on request from the corresponding author, as the study is still ongoing.

## Supplementary Materials

The following supporting information can be downloaded at: https://www.mdpi.com/article/10.3390/life15111726/s1, Figure S1: Isotype controls performed for immunofluorescence staining on whole-mounted rat retinas. Figure S2: Escitalopram increases the content of Connexin 36.

## Abbreviations

The following abbreviations are used in this manuscript:

BDNF	Brain-derived neurotrophic factor
CNS	Central nervous system
CXs	Connexins
ERG	Electroretinography
EVC	Episcleral vein cauterization
GCL	Ganglion cell layer
IOP	Intraocular pressure
MFA	Meclofenamic acid
NeuN	Neuronal-Nuclei
OPs	Oscillatory potentials
PhNR	Photopic negative response
RBPMS	RNA-binding protein with multiple splicing
RGCs	Retinal ganglion cells
SSRIs	Selective serotonin reuptake inhibitors
