# Supplementary figures and images for: Serotonin Signaling Pathway Modulation Affects Retinal Neuron Survival in Experimental Model of Retinal Ischemia
Source: Life (Basel). 2025 Nov 8;15(11):1726. doi: 10.3390/life15111726 (PMC12653536; doi:10.3390/life15111726)

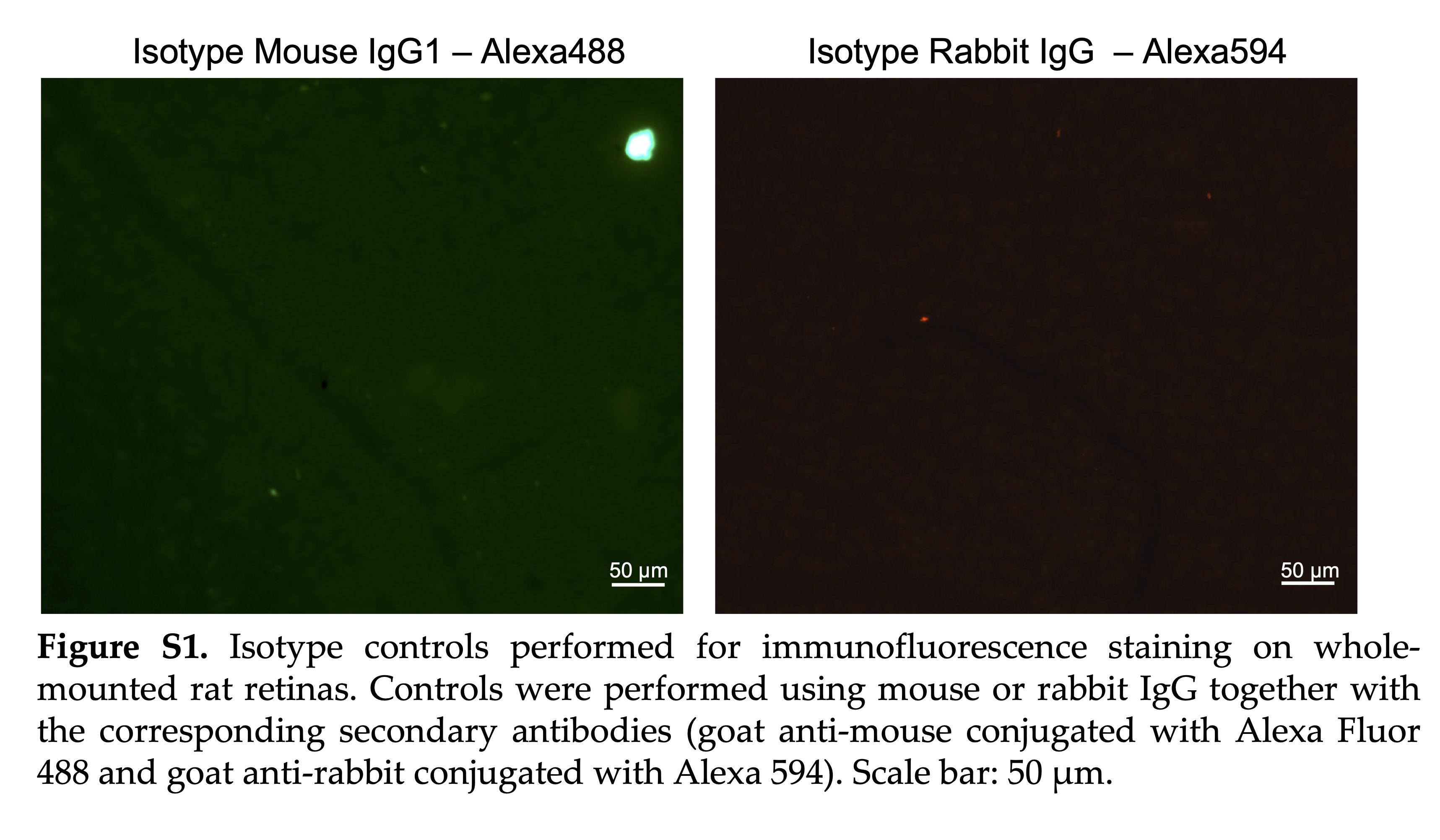

Supplement: Supplementary file 1 [file life-15-01726-s001.zip › Figure S1.tiff]
